# External validation of the HALP score for predicting in-hospital and long-term outcomes in acute heart failure: a retrospective cohort study

**DOI:** 10.3389/fcvm.2026.1958343

**Published:** 2026-10-30

**Authors:** Mehmet Zafer Aydın, Mustafa Gök, Mehmet Kamil Teber, Zülfiye Kuzu, Ziya Şimşek

**Affiliations:** Department of Cardiology, Kayseri City Training and Research Hospital, Kayseri, Türkiye

**Keywords:** external validation, HALP score, heart failure, mortality, prognosis, risk stratification

## Abstract

**Background:**

The hemoglobin–albumin–lymphocyte–platelet (HALP) score integrates nutritional, inflammatory, and hematologic status and has been proposed as a prognostic marker in acute heart failure (AHF), but external validation is limited.

**Methods:**

We retrospectively analyzed 197 consecutive patients hospitalized with AHF at a tertiary center. HALP was calculated at admission, and the pre-specified outcomes were in-hospital mortality, prolonged hospital stay (>7 days), all-cause mortality over follow-up, and heart-failure rehospitalization. Discrimination was assessed by the area under the receiver-operating-characteristic curve (AUC) with DeLong 95% confidence intervals (CIs), and associations were tested with crude and age/sex-adjusted logistic regression (in-hospital outcomes) and Cox regression (mortality over follow-up), using both data-derived (Youden) and pre-specified HALP thresholds.

**Results:**

The cohort had a median age of 71 years (30.5% female) and a median follow-up of 15.5 months; in-hospital mortality was 12.7%, prolonged stay 19.4%, and all-cause mortality 31.0%. HALP did not discriminate any outcome (AUC 0.53 [95% CI 0.41–0.65] for in-hospital mortality, 0.47 [0.36–0.59] for prolonged stay, and 0.57 [0.48–0.66] for all-cause mortality; all CIs included 0.50). A Youden-optimized threshold appeared associated with mortality in logistic regression (adjusted odds ratio 2.02, 95% CI 1.02–4.00), but the signal vanished under pre-specified thresholds (median-split adjusted odds ratio 1.23, 0.67–2.28) and in time-to-event analysis (adjusted hazard ratio per 1-SD decrease 1.04, 0.78-1.38; Youden threshold 1.75, 0.97-3.16; log-rank across quartiles p = 0.15). In the same patients, the established EHMRG and MAGGIC scores discriminated mortality (AUC 0.71 and 0.65; CIs excluding 0.50), whereas HALP correlated only with severity markers (NYHA class rho = −0.31; NT-proBNP rho = −0.16; CRP rho = −0.17).

**Conclusion:**

In this independent cohort, HALP reflected disease severity but did not predict outcomes once cutoff-optimization bias was removed, and should not be used as a stand-alone risk-stratification tool in AHF.

## Introduction

Heart failure (HF) remains a leading cause of hospitalization and death, and risk stratification at admission informs both intensity of care and discharge planning. Simple biomarkers derived from routine complete blood counts and basic chemistry are attractive because they are inexpensive, universally available, and reproducible.

The HALP score, combining hemoglobin, albumin, lymphocyte count, and platelet count, was originally described in oncology as a composite index of nutritional and immune–inflammatory status (1). Lower values reflect anemia, hypoalbuminemia, lymphopenia, or a heightened inflammatory state. The score has since been extended to cardiovascular disease, where it has been evaluated across diverse settings — for example, predicting outcomes after percutaneous coronary intervention in elderly patients with acute myocardial infarction (2), respiratory and intensive-care outcomes after minimally invasive valve surgery (3), and postoperative adverse events after coronary artery bypass grafting (4) — reflecting its appeal as a simple, routinely available index. In heart failure specifically, our group previously reported in a single-center study that a low HALP score was associated with prolonged hospitalization and adverse outcomes in acute heart failure (AHF) (5), although its discriminative accuracy for prolonged stay was modest (reported AUC 0.51); other single-center cohorts have associated low HALP with in-hospital and short-term mortality in AHF (6, 7). Large database analyses have similarly linked lower HALP to higher mortality and longer stay; in a 4,286-patient MIMIC-IV cohort, higher HALP was associated with reduced short- and long-term mortality, though the effect sizes were small and the authors cautioned that HALP should complement rather than replace existing risk tools (8).

External validation in an independent population is an essential step before a prognostic marker enters clinical use, and is particularly important when the original report relied on a data-derived cutoff (9). We therefore examined whether the HALP score predicts in-hospital and long-term outcomes in a separate AHF cohort, and specifically whether any association is robust to the choice of threshold and to the analytical approach.

## Materials and methods

### Study design and population

We conducted a single-center, retrospective cohort study of consecutive adult patients (≥18 years) hospitalized with a primary diagnosis of acute heart failure in the Department of Cardiology of Kayseri City Training and Research Hospital between January 2019 and September 2025. Patients were identified from the hospital information system using ICD-10 codes for heart failure (I50.x) and were included when the discharge diagnosis of acute heart failure — new-onset or decompensated chronic heart failure — was confirmed on chart review according to the 2021 ESC Heart Failure Guidelines (10), i.e., typical symptoms and signs of congestion together with objective evidence of cardiac dysfunction on echocardiography and/or an elevated natriuretic peptide level. Patients were excluded if they had an acute coronary syndrome as the primary diagnosis (*n* = 123); had active malignancy (*n* = 13) or hematologic disease (*n* = 2); had active infection or sepsis (*n* = 23); had received a blood transfusion within the preceding month (*n* = 4); or had incomplete admission laboratory data for hemoglobin, albumin, lymphocyte or platelet count (*n* = 23). Of 386 patients screened, 188 were excluded for these reasons and one record contained no analyzable data, leaving 197 patients in the final cohort (Supplementary Figure S1). Demographic characteristics, comorbidities, NYHA class, admission laboratory values, medications, and validated risk scores [MAGGIC (11) and EHMRG (12)] were extracted from the medical record. The study is reported in accordance with the STROBE statement for observational studies (13).

The study was approved by the Kayseri City Training and Research Hospital Non-Interventional Clinical Research Ethics Committee (decision no. 685, dated 2 December 2025) and was conducted in accordance with the Declaration of Helsinki. Because the study used only retrospective, de-identified archival records, the requirement for individual informed consent was waived by the committee.

### HALP score calculation

The HALP score was calculated from admission values as HALP = Hemoglobin (g/L) × Albumin (g/L) × Lymphocyte count (/L) ÷ Platelet count (/L), consistent with the index study (5). Hemoglobin in g/dL was converted to g/L; albumin was recorded in g/L; lymphocyte and platelet counts were expressed as ×10⁹/L. One implausible lymphocyte value (a misplaced decimal) was set to missing, leaving 196 evaluable HALP scores.

### Outcomes

The pre-specified outcomes were: (i) in-hospital mortality; (ii) prolonged hospital stay, defined *a priori* as length of stay >7 days to match the index study (≥7 days was examined in sensitivity analysis); (iii) all-cause mortality over the entire follow-up period, analyzed as a time-to-event outcome; and (iv) heart-failure rehospitalization. Heart-failure rehospitalization was assessed among the 172 patients discharged alive from the index hospitalization, because this outcome is not defined for patients who died in hospital. As an additional, *post hoc* analysis, we examined post-discharge mortality — all-cause death after discharge among patients discharged alive — to assess whether any association with HALP was confined to the post-discharge period. Follow-up time was defined as the interval from index admission to death or last contact; for patients who died during the index hospitalization, the length of stay was used as survival time. Outcome codes were decoded from the original data-set value labels; all-cause mortality combined in-hospital deaths with deaths recorded during follow-up.

### Statistical analysis

Continuous variables are summarized as median (interquartile range) and compared with the Mann–Whitney U test; categorical variables as *n* (%) and compared with the *χ*^2^ test. Discrimination was quantified by the area under the receiver-operating-characteristic curve (AUC) with its 95% confidence interval (CI) calculated by the DeLong method (14); because lower HALP indicates poorer status, the ROC direction was set so that lower values corresponded to the event. The association of HALP with each outcome was tested under four definitions: a Youden-index–optimized cutoff derived for each outcome in the same data, a pre-specified median split, the lowest quartile, and the standardized continuous score (per 1-SD decrease). For the in-hospital outcomes (in-hospital mortality and prolonged stay), for which every patient had a complete and equal observation window, binary logistic regression was used; for mortality over follow-up and post-discharge mortality, which are time-to-event outcomes with unequal follow-up, Cox proportional-hazards regression was used, with the proportional-hazards assumption checked by scaled Schoenfeld residuals. Logistic-regression odds ratios for the follow-up outcomes are also reported for comparability with the index study. All models were fitted unadjusted (crude) and adjusted for age and sex; further adjustment was not attempted because of the limited number of events. Survival was displayed by Kaplan–Meier curves according to HALP quartile and compared with the log-rank test; a median-split comparison was performed as a sensitivity analysis. Event rates across HALP quartiles were compared with the *χ*^2^ test and a test for linear trend. Construct validity was assessed by Spearman rank correlation of HALP with severity markers. A two-sided *p* < 0.05 was considered significant. Analyses were performed with IBM SPSS Statistics for Windows, version 22.0 (IBM Corp., Armonk, NY, USA) and Python 3.11 (statsmodels 0.14, lifelines 0.30).

## Results

### Baseline characteristics

The cohort had a median age of 71 years and 30.5% were women, with a high burden of hypertension (76.1%), coronary artery disease (61.4%), and diabetes (40.1%). Median follow-up was 15.5 months. Baseline characteristics stratified by all-cause mortality are shown in Table 1. Survivors and decedents did not differ significantly in age, sex, HALP components, or the HALP score itself, whereas the established MAGGIC and EHMRG risk scores were significantly higher among those who died (*p* = 0.009 and *p* < 0.001).

**Table 1:** Baseline characteristics of the cohort overall and by all-cause mortality.

Characteristic	Overall (*n* = 197)	Survivors (*n* = 136)	Died (*n* = 61)	p
Age, years	71 (63–77)	71 (64–77)	71 (62–79)	0.68
Female	60 (30.5)	46 (33.8)	14 (23.0)	0.17
Body-mass index, kg/m^2^	26.2 (24.2–29.8)	26.6 (24.2–30.2)	25.9 (24.2–29.4)	0.47
Diabetes mellitus	79 (40.1)	57 (41.9)	22 (36.1)	0.54
Hypertension	150 (76.1)	99 (72.8)	51 (83.6)	0.14
Coronary artery disease	121 (61.4)	79 (58.1)	42 (68.9)	0.20
Atrial fibrillation	69 (35.0)	49 (36.0)	20 (32.8)	0.78
Chronic kidney disease	49 (24.9)	33 (24.3)	16 (26.2)	0.91
COPD	69 (35.0)	45 (33.1)	24 (39.3)	0.49
NYHA class	4 (3–4)	4 (3–4)	4 (3–4)	0.10
HALP components and score				
Hemoglobin, g/dL	12.9 (11.4–14.3)	13.0 (11.6–14.5)	12.5 (11.2–13.9)	0.23
Albumin, g/L	38 (34–40)	38 (35–40)	38 (33–41)	0.68
Lymphocytes, ×10⁹/L	1.3 (0.9–2.1)	1.4 (0.9–2.2)	1.3 (0.7–1.8)	0.13
Platelets, ×10⁹/L	234 (178–295)	243 (177–300)	225 (178–279)	0.48
HALP score	28.4 (17.1–41.8)	29.3 (18.5–44.6)	26.0 (16.5–35.0)	0.11
Other laboratory values				
Creatinine, mg/dL	1.3 (1.0–1.7)	1.2 (1.0–1.6)	1.4 (1.0–1.9)	0.11
NT-proBNP, pg/mL	5,883 (2,592–15,340)	4,672 (2,525–17,648)	7,061 (3,400–14,426)	0.44
CRP, mg/L	17.5 (7.6–49.2)	16.9 (7.3–45.4)	24.6 (9.4–54.9)	0.12
MAGGIC risk score (*n* = 121)	30 (24–33)	29 (23–32)	33 (28–35)	0.009
EHMRG score (*n* = 118)	166 (133–200)	156 (127–184)	189 (167–210)	<0.001

Values are median (IQR) or *n* (%). *P* values from Mann–Whitney U or *χ*^2^ tests. MAGGIC and EHMRG scores were available in 121 and 118 patients, respectively.

### Outcome rates

In-hospital mortality was 12.7% and prolonged stay (>7 days) 19.4%. In-hospital mortality was comparable to the index study (9.5%), whereas prolonged stay was less frequent than in the index study (38.7%), reflecting the shorter hospital stays in our cohort (median 5 days). Over follow-up, all-cause mortality reached 31.0%; among the 172 patients discharged alive, heart-failure rehospitalization occurred in 51.2% and post-discharge death in 20.9% (Table 2).

**Table 2:** Outcome frequencies.

Outcome	Events/n	Rate (%)
In-hospital mortality	25/197	12.7
Prolonged stay >7 days	38/196	19.4
Prolonged stay ≥7 days (sensitivity)	56/196	28.6
All-cause mortality over follow-up	61/197	31.0
Heart-failure rehospitalization^a^	88/172	51.2
Post-discharge mortality^a^ (additional analysis)	36/172	20.9

a Assessed among the 172 patients discharged alive from the index hospitalization.

### HALP discrimination and the effect of threshold choice

The HALP score did not discriminate any outcome: every AUC 95% CI included 0.50 (Table 3, Figure 1A). The Youden-optimized cutoffs were outcome-specific and ranged from 14.2 to 41.8 (Table 3). In logistic regression, the Youden threshold produced an apparently significant association with all-cause mortality (crude OR 2.04, 95% CI 1.03–4.01; age- and sex-adjusted OR 2.02, 1.02–4.00) and with post-discharge mortality (adjusted OR 2.44, 1.13–5.29). However, these associations were entirely dependent on the optimized cutoff. Under the pre-specified median split (adjusted OR for all-cause mortality 1.23, 95% CI 0.67–2.28), the lowest quartile (1.56, 0.78–3.12), or the continuous standardized score (1.04 per 1-SD decrease, 0.76–1.44), no association remained, and crude and adjusted estimates were virtually identical for every definition (Table 3, Figure 1B). Event rates showed no monotonic gradient across HALP quartiles (all-cause mortality 36.7%, 28.6%, 38.8%, 18.4% from lowest to highest quartile; *p* for trend = 0.13; Figure 1C).

**Table 3:** Discrimination of the HALP score for each outcome and odds ratios for low HALP under data-derived and pre-specified threshold definitions.

Outcome (n/events)	AUC (95% CI)	Threshold	Crude OR (95% CI)	p	Adjusted OR (95% CI)	p
In-hospital mortality (196/25)	0.53 (0.41–0.65)	Youden (≤41.8)	2.70 (0.77–9.45)	0.12	2.71 (0.77–9.51)	0.12
		Median (≤28.4)	0.76 (0.33–1.77)	0.52	0.77 (0.33–1.79)	0.54
		Lowest quartile (≤16.9)	1.19 (0.47–3.06)	0.71	1.28 (0.50–3.31)	0.61
		Per 1-SD decrease	1.29 (0.71–2.35)	0.41	1.31 (0.71–2.42)	0.39
Prolonged stay >7 d (195/37)	0.47 (0.36–0.59)	Youden (≤14.2)	1.90 (0.76–4.74)	0.17	2.01 (0.79–5.10)	0.14
		Median (≤28.4)	0.92 (0.45–1.89)	0.83	0.93 (0.45–1.91)	0.84
		Lowest quartile (≤16.9)	1.34 (0.60–2.95)	0.47	1.36 (0.61–3.03)	0.45
		Per 1-SD decrease	0.84 (0.61–1.14)	0.26	0.85 (0.62–1.15)	0.29
All-cause mortality (196/60)	0.57 (0.48–0.66)	Youden (≤34.4)	2.04 (1.03–4.01)	0.040	2.02 (1.02–4.00)	0.043
		Median (≤28.4)	1.21 (0.66–2.23)	0.54	1.23 (0.67–2.28)	0.50
		Lowest quartile (≤16.9)	1.45 (0.73–2.87)	0.28	1.56 (0.78–3.12)	0.21
		Per 1-SD decrease	1.04 (0.76–1.42)	0.83	1.04 (0.76–1.44)	0.79
Post-discharge mortality^a^ (171/35)	0.59 (0.48–0.70)	Youden (≤19.2)	2.43 (1.13–5.24)	0.023	2.44 (1.13–5.29)	0.023
		Median (≤28.4)	1.59 (0.75–3.39)	0.23	1.60 (0.75–3.41)	0.23
		Lowest quartile (≤16.9)	1.55 (0.68–3.52)	0.29	1.62 (0.71–3.72)	0.25
		Per 1-SD decrease	0.95 (0.68–1.33)	0.76	0.96 (0.68–1.34)	0.79
HF rehospitalization^a^ (171/88)	0.47 (0.38–0.56)	Youden (≤14.9)	1.57 (0.69–3.58)	0.29	1.57 (0.68–3.63)	0.29
		Median (≤28.4)	0.85 (0.46–1.54)	0.59	0.85 (0.46–1.55)	0.59
		Lowest quartile (≤16.9)	1.05 (0.52–2.11)	0.89	1.02 (0.51–2.07)	0.95
		Per 1-SD decrease	1.00 (0.75–1.33)	0.99	1.01 (0.76–1.35)	0.93

AUC with DeLong 95% CI; ROC direction set so that lower HALP indicates the event. Odds ratios from binary logistic regression for HALP at or below the stated threshold versus above it, or per 1-SD (33.7-point) decrease in the continuous score; adjusted models include age and sex. Youden cutoffs were derived separately for each outcome in the same data.

a Assessed among patients discharged alive.

**Figure 1:**
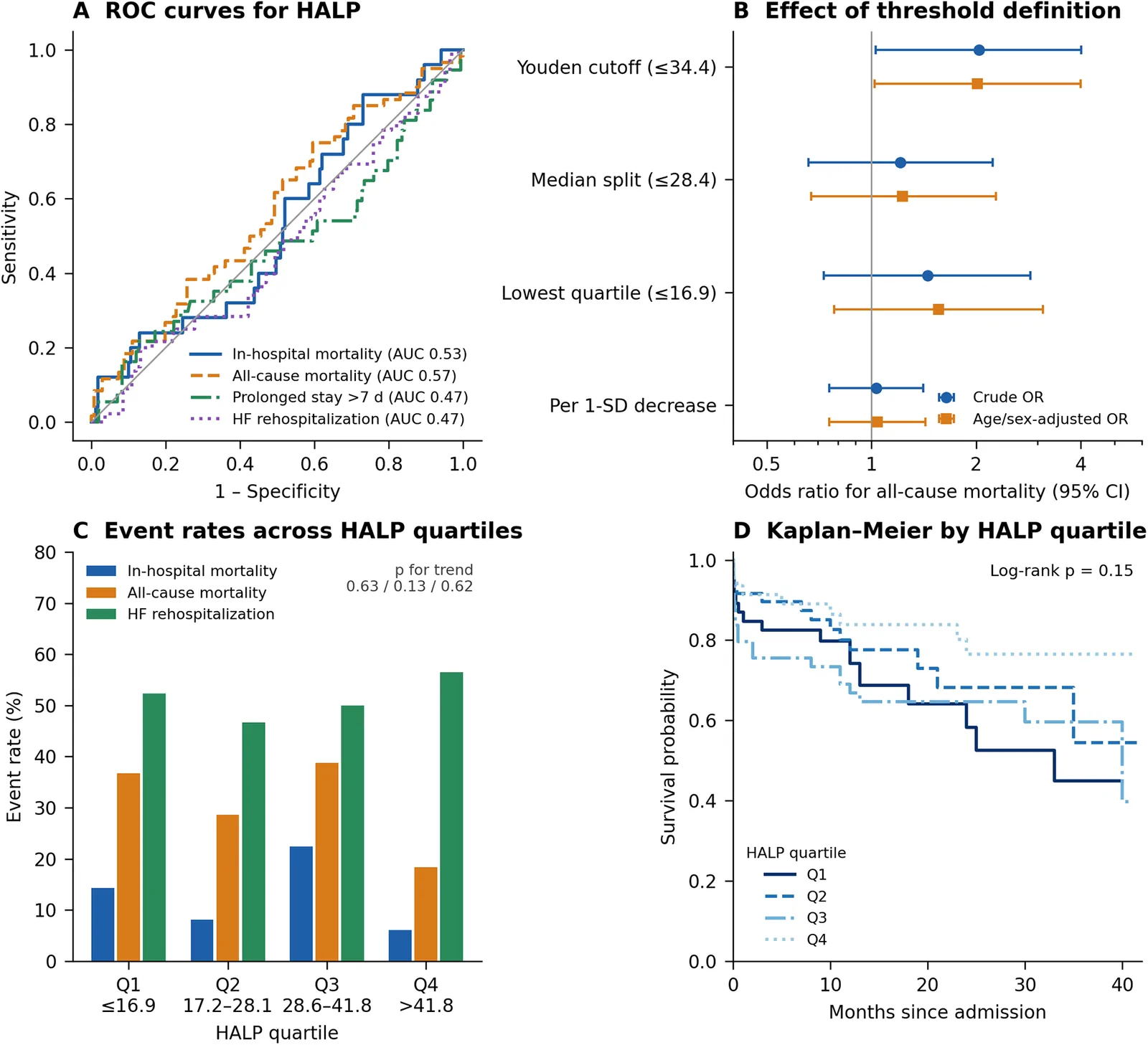
External validation of the HALP score (*n* = 196 with evaluable HALP). **(A)** ROC curves for in-hospital mortality, all-cause mortality, prolonged stay, and HF rehospitalization; all AUC 95% CIs (DeLong) include 0.50. **(B)** Crude and age/sex-adjusted odds ratios for all-cause mortality under the Youden-optimized threshold and the pre-specified definitions (median split, lowest quartile, per 1-SD decrease); the association collapses toward the null once the cutoff is pre-specified. **(C)** Event rates across HALP quartiles for in-hospital mortality, all-cause mortality and HF rehospitalization (*p* for trend 0.63, 0.13 and 0.62); rehospitalization among patients discharged alive. **(D)** Kaplan–Meier survival by HALP quartile (*n* = 189; log-rank *p* = 0.15).

### Time-to-event analysis of mortality

Time-to-event analysis (189 patients with recorded follow-up time; 58 deaths) confirmed the absence of an association (Table 4). In Cox regression, HALP was not associated with all-cause mortality when modeled continuously (adjusted HR per 1-SD decrease 1.04, 95% CI 0.78–1.38), by median split (1.21, 0.72–2.04) or by lowest quartile (1.58, 0.89–2.80). Notably, even the Youden-optimized threshold — the only definition that reached nominal significance in the logistic model — was not significantly associated with mortality in the Cox model (adjusted HR 1.75, 95% CI 0.97–3.16, *p* = 0.063). The proportional-hazards assumption was satisfied (Schoenfeld residual test, *p* = 0.87). Kaplan–Meier survival did not differ across HALP quartiles (log-rank *p* = 0.15; Figure 1D), nor by median split (log-rank *p* = 0.54); estimated 12-month survival was 74%, 78%, 67% and 84% from the lowest to the highest quartile, with no ordered gradient. Results for post-discharge mortality were similar (Table 4).

**Table 4:** Cox proportional-hazards regression for HALP and mortality over follow-up.

Outcome (n/events)	HALP definition	Crude HR (95% CI)	p	Adjusted HR (95% CI)	p
All-cause mortality (189/58)	Per 1-SD decrease	1.02 (0.77–1.35)	0.89	1.04 (0.78–1.38)	0.78
	Median (≤28.4)	1.17 (0.70–1.97)	0.54	1.21 (0.72–2.04)	0.47
	Lowest quartile (≤16.9)	1.47 (0.83–2.59)	0.19	1.58 (0.89–2.80)	0.12
	Youden (≤34.4)	1.75 (0.97–3.15)	0.062	1.75 (0.97–3.16)	0.063
Post-discharge mortality^a^ (164/33)	Per 1-SD decrease	0.92 (0.67–1.26)	0.59	0.95 (0.69–1.30)	0.73
	Median (≤28.4)	1.60 (0.80–3.19)	0.19	1.69 (0.84–3.41)	0.14
	Lowest quartile (≤16.9)	1.70 (0.81–3.58)	0.16	1.82 (0.85–3.92)	0.12

Hazard ratios for low HALP (at or below the stated threshold versus above it) or per 1-SD decrease; adjusted models include age and sex. Follow-up time from index admission to death or last contact (length of stay for in-hospital deaths); seven patients without recorded follow-up time were excluded.

a Among patients discharged alive, from discharge to death or last contact.

### Comparison with established risk scores

To confirm that the null findings were not simply due to limited statistical power, we evaluated two established scores in the same patients (11, 12). Both discriminated all-cause mortality with confidence intervals excluding 0.50 — EHMRG (AUC 0.71, 95% CI 0.61–0.80; *n* = 118) and MAGGIC (AUC 0.65, 0.54–0.76; *n* = 121) — whereas HALP did not (0.57, 0.48–0.66). The cohort therefore contained a detectable prognostic signal that HALP failed to capture.

### Construct validity

Despite its lack of predictive value, HALP behaved coherently as a severity marker: lower HALP correlated with higher NYHA class (rho=−0.31, *p* < 0.001), higher NT-proBNP (rho=−0.16, *p* = 0.03), and higher CRP (rho=−0.17, *p* = 0.02). It did not correlate with the MAGGIC or EHMRG scores.

## Discussion

In this independent cohort of patients hospitalized with acute heart failure, the HALP score did not predict in-hospital mortality, prolonged hospitalization, or long-term mortality. The only nominally significant associations emerged from a threshold optimized within the same data and disappeared when a pre-specified cutoff was applied or when mortality was analyzed appropriately as a time-to-event outcome — the hallmark of cutoff-optimization bias. This pattern mirrors our group's earlier report (5), in which the reported discrimination for prolonged stay was poor (AUC 0.51) yet a dichotomized cutoff yielded a large odds ratio. The observation that the Youden cutoff itself varied from 14.2 to 41.8 depending on the outcome to which it was fitted illustrates the instability of such thresholds. Our data suggest that dichotomized associations of this kind are fragile and may not generalize.

Importantly, the null result is unlikely to be explained by insufficient signal or power alone. Within the same patients, the EHMRG and MAGGIC scores discriminated mortality with confidence intervals excluding chance. The failure was therefore specific to HALP rather than a property of the cohort. At the same time, HALP correlated with congestion and inflammation markers, indicating that it does measure aspects of disease severity — it simply does not translate that information into accurate outcome prediction in AHF, where hemodynamic and neurohormonal factors dominate prognosis.

Why might HALP carry prognostic information in other cardiovascular populations but not here? Positive reports exist for elderly patients undergoing percutaneous coronary intervention for acute myocardial infarction (2), for respiratory and intensive-care outcomes after minimally invasive valve surgery (3), and for postoperative adverse events after coronary artery bypass grafting, where a postoperative HALP ≤10.93 identified patients with complications with reported sensitivity and specificity of 97% and 99% (4). Several differences between those settings and ward-based AHF are relevant. First, the timing and meaning of the measurement differ: a postoperative HALP captures the acute surgical inflammatory response, hemodilution and blood loss, and perioperative nutritional depletion — processes that are proximate causes of postoperative complications — whereas an admission HALP in AHF reflects a chronic and relatively stable background of anemia, hypoalbuminemia and lymphopenia that is present in most patients with advanced disease. Second, the relative weight of the pathways HALP indexes differs. In surgical and acute coronary populations, inflammatory burden and nutritional reserve are strong determinants of infection, wound healing and recovery; in AHF, prognosis is driven principally by congestion, renal function, neurohormonal activation and the capacity to respond to decongestive therapy, none of which HALP measures. Third, the dynamic range of the score is compressed in AHF: in our cohort the median NYHA class was 4 and anemia, hypoalbuminemia and lymphopenia were common, so a low HALP discriminated poorly between patients who did and did not go on to die. Fourth, the outcomes differ in horizon and mechanism — short-term postoperative complications versus mortality and rehospitalization over more than a year — and a marker of acute physiological reserve need not predict long-term events. Finally, several of the positive studies, like the index study, relied on thresholds derived within the same data set, and the exceptionally high sensitivity and specificity reported for the postoperative HALP cutoff after bypass surgery (4) would themselves benefit from external validation. Our study provides precisely that type of evaluation for AHF.

These observations can also be reconciled with the larger positive literature in heart failure. Even in a 4,286-patient MIMIC-IV cohort (8), higher HALP was associated with only modest reductions in mortality (fully adjusted hazard ratio ≈0.92–0.93 per 10 points), and the investigators themselves concluded that HALP should complement rather than replace existing risk tools. Very large samples can render such small effects statistically significant without conferring clinically useful discrimination at the bedside. Our findings extend this by showing that, in a smaller ward-based cohort using pre-specified thresholds and time-to-event methods, the modest HALP signal does not translate into meaningful risk stratification. Differences in case-mix — an intensive-care population in the database studies and coronary-care-unit populations in some single-center reports (7) versus general ward admissions here — may further contribute to the divergent conclusions.

These findings carry a broader methodological message. Prognostic thresholds derived by ROC/Youden optimization are optimistically biased and should be reported alongside pre-specified or cross-validated cutoffs and external confirmation before clinical adoption (9). For HALP specifically, the current evidence does not support its use as a stand-alone risk-stratification tool in acute heart failure.

### Limitations

This was a single-center, retrospective study, and residual confounding and selection bias cannot be excluded. Multivariable models were adjusted only for age and sex because the number of in-hospital events limited the number of covariates that could be included; more extensive adjustment would not, however, be expected to create an association that is absent in crude analysis. HALP was measured only at admission, so we could not assess trajectory. Follow-up duration varied — addressed by time-to-event analysis — and seven patients without a recorded follow-up time could not be included in the survival analyses; some laboratory and follow-up fields were incomplete, and the MAGGIC and EHMRG scores were available in a subset of patients. The number of hard events, while adequate to detect the discrimination shown by EHMRG and MAGGIC, limits precision for small effects; nevertheless, the confidence intervals argue against a clinically meaningful missed association. Finally, outcome definitions were reconstructed from data-set value labels and may differ from those of the index study.

## Conclusion

In conclusion, the HALP score reflected heart-failure severity but did not independently predict in-hospital, length-of-stay, or long-term outcomes in this external cohort once cutoff-optimization bias was removed and mortality was analyzed as a time-to-event outcome, while established risk scores performed as expected in the same patients. These results temper enthusiasm for HALP as a prognostic tool in acute heart failure and underscore the need for pre-specified thresholds and external validation of biomarker cutoffs.

## Data Availability

The raw data supporting the conclusions of this article will be made available by the authors, without undue reservation.

## Supplementary material

Supplementary Figure S1 Patient flow diagram: 386 patients screened; 188 excluded (acute coronary syndrome as primary diagnosis, *n* = 123; active malignancy, *n* = 13; hematologic disease, *n* = 2; active infection/sepsis, *n* = 23; blood transfusion within the preceding month, *n* = 4; incomplete admission laboratory data, *n* = 23); one record without analyzable data; 197 patients analyzed (196 with evaluable HALP; 172 discharged alive; 189 with recorded follow-up time for survival analysis).
